# Party preferences for climate policy and the renewable energy transition in Spain’s multilevel democracy

**DOI:** 10.1038/s44168-024-00183-5

**Published:** 2024-10-31

**Authors:** Joan Enguer

**Affiliations:** Institute of Political Science, Heidelberg University, Heidelberg, Germany

**Keywords:** Social sciences, Politics, Climate change

## Abstract

The growing influence of regional governments in shaping climate policy and driving the renewable energy transition in multilevel democracies like Spain provides incentives for parties in favor of decentralization to emphasize these issues. Recent research has shown that such parties act as climate pioneers at the regional tier of government, driven by their desire to assert stronger subnational authority. To investigate these dynamics at the national level, this article examines the manifestos of the parties that won seats in the 2016, 2019, and 2023 Spanish national elections. The empirical findings suggest that parties are more likely to prioritize climate change and the renewable energy transition if they are pro-decentralization. By emphasizing how multilevel governance strengthens these priorities through party competition and the quest for regional autonomy, this article fills an important gap spanning decentralization and policy preferences related to climate and renewable energy.

## Introduction

In 2021, Spain enacted the Climate Change and Energy Transition Law (Law 7/2021 of 20 May), which consolidates the country’s commitment to climate change and the energy transition for the coming decades. This emblematic law, which was two years in the making, is the culmination of Spain’s extensive regulatory trajectory in these areas. This trajectory has been favored by the decentralized institutional framework established by the Spanish Constitution under the name of the ‘State of Autonomies’. The evolution of this model has cemented the shared nature of competences in the areas of climate change and the energy transition between the central State and the regional governments, also known as Autonomous Communities (ACs)^1^. As a result, the ACs have assumed an increasingly prominent role in these issues, participating in certain national initiatives and introducing their own policies^2^. There are notable examples in several regions, such as the enactment of Law 16/2017 by Catalonia, the approval of Law 8/2018 by Andalusia, or the adoption of Law 10/201 by the Balearic Islands^3^.

In this vein, extensive research on Climate Federalism has identified several benefits of decentralized political systems in addressing climate-related challenges^4,5^, which can also catalyze the renewable energy transition (RET)^6,7^. Significantly, the distributed responsibilities and decision-making inherent in devolved governance promote experimentation, mutual learning, and healthy competition among subnational entities^8,9^. This is because decentralized systems provide multiple levels for developing and testing new climate and energy policies. The result is a higher likelihood of identifying successful strategies, which can then spread horizontally across regions or vertically to influence national plans^10,11^.

Moreover, decentralized states create an opportunity structure in which constituent units can initiate their own climate action programs in the absence of central government action. This phenomenon, known as ‘compensatory federalism,’ was evident during periods of federal climate inaction in the United States and has parallels in Spain, where regional laws in Catalonia, the Balearic Islands, and Andalusia have paved the way for climate leadership^3,12,13^. This mechanism is especially salient in light of the potential benefits regions can gain regarding the deployment of renewable energy installations throughout their territories. Indeed, certain subnational governments may prioritize the transformation of regional energy economies using renewables that are available locally, hence fostering regional advantages^14^. In this way, the RET offers opportunities and incentives for subnational governments with adequate competencies to leverage their potential in developing their own energy strategies and policies independently from the central government^15^.

Additionally, decentralized governance enhances the capacity of regional parties to adapt policies to the context^16^. Devolving autonomy of climate policy to decentralized entities empowers them to integrate local knowledge, capacities, and resources when crafting solutions that fit unique conditions of the environment, society, and economy^5^. Put differently, regional authorities are better able to handle energy transitions when they have a say in the creation of broad-based renewable energy plants within the expanding decentralized energy grids accompanying the RET^17^. Empirical evidence supports such a view, showing that decentralized energy models not only improve electricity access in rural areas^18^ but also reduce CO_2_ emissions significantly^19^. This derives from their efficacy in meeting simultaneously the increasing demand for renewable energy sources and demands for land and protection of the environment^20^.

In the same manner, decentralization brings policymaking processes closer to citizens, enhancing both public participation and the public acceptance of climate and energy policies^5,9^. It thereby contributes to the establishment of ‘energy democracy’ systems with active citizens who are engaged with and take responsibility for energy production and consumption^21,22^. Such systems foster greater societal commitment to shifting away from fossil fuel-based energy, promoting renewable energy generation, and potentially claiming ownership and management of energy infrastructure^23^. In this context, the role of renewable energy cooperatives (REC) stands out. These co-operatives serve as testbeds for adapting low-carbon energy technologies to local conditions and needs, thus playing a crucial role in the energy transition^24,25^. The historic proliferation of RECs in countries such as the UK, Belgium, Denmark, and Germany has coincided with support schemes aimed at boosting RET^26^. In addition, these organizations are increasingly involved in energy governance, particularly at the subnational level^27^.

The academic evidence in favor of decentralizing climate change and RET responsibilities has been paralleled by an expansion of the role of subnational governments in these areas^11,28–31^. This trend towards regional empowerment encourages pro-decentralization parties, such as the non-state parties (NSWP), to prioritize these issues^32–34^. In light of this, some scholars have examined how territorial politics influence climate and energy agendas, highlighting a form of nationalism centered on environmental issues that advocates for comprehensive climate policies^35^. Some related studies suggest that the RET, with its low carbon attributes, can strengthen arguments and promote nationalist agendas within this evolving framework of sustainable nationalism^36^.

This new concept, coined as Green Nationalism^37–39^, is present, for example, in regions like the Vauban district, where researchers have observed a revitalization of national sentiments based on environmental defense^40^. This resurgence is attributed to the pride the region takes in Germany’s example of adapting to the challenges posed by the climate crisis. Likewise, the literature reveals that minority nationalist parties, such as We Make Corsica/For Corsica^35,41^, and the Galician National Bloc (BNG)^35^^,^, have been weaving together environmentalism and autonomy in their campaigns. They increasingly emphasize climate change as a phenomenon requiring locally adapted solutions.

Similarly, the British Greens and the Scottish Green Party are known to question the nation-state to which they belong and propose an original, regionalist project^42^. In the case of Scotland, these findings align with previous research linking the strength of substate climate ambitions with the substantial emphasis they place on regional governmental autonomy^43^. This correlation is reinforced by the influence of strong sub-state nationalist parties, which actively work to maintain and reinforce regional divisions.

Some minority nationalist parties, such as the Scottish National Party and the Republican Left of Catalonia (ERC), are known to mix traditional, preservationist, nationalist ideals with contemporary policies on climate change. They do so through their assertions in manifestos, posters, policy briefs, and flyers, as well as through the promotion of green policies and bill proposals^39^. In the Basque Country and Catalonia, recent studies have documented how nationalist parties strategically incorporate climate change concerns into their assertion of claims, narratives, and frameworks as integral parts of their nationalist endeavors^35,44^. Regarding Catalonia, other research indicates that parties more aligned with decentralization tend to place greater emphasis on climate policy and adopt more pro-climate positions. They do this to compete for political authority, in addition to addressing environmental concerns^45^.

Existing literature highlights the significant benefits of decentralized political systems in driving climate initiatives^4,5^ and the transition to renewable energy sources^6,7^. It is, therefore, reasonable to expect parties advocating decentralization to take a pioneering role in these issues. Given the advantages of decentralization in addressing climate and energy challenges effectively, these issues align more naturally with the core objectives of parties that support the devolution of powers to lower levels of government. Additionally, in accordance with the principles of Green Nationalism, such actors may be incentivized to emphasize climate policy and the RET, as these areas enable subnational authorities to formulate and implement their own policies and strategies^37–39^. Specifically, in the context of the RET, building new facilities for generating renewable energy (e.g., solar photovoltaic, wind turbines, hydroelectric power, biomass, or geothermal power plants) would create a landscape of decentralized production, which is particularly appealing to parties seeking greater regional autonomy. Thus, the potential of the RET for facilitating a shift toward regional energy independence would incentivize pro-decentralization parties, such as NSWPs, to advocate for this issue. In other words, pro-decentralization parties would be more likely to position themselves at the forefront of these matters as a strategic move to challenge the central government and enhance subnational authority.

Building on the anticipated link between political parties’ advocacy for decentralization and their focus on climate and RET issues, I propose two hypotheses:

**Hypothesis 1**: The salience of climate content in national party manifestos increases the more pro-decentralization the party is.

**Hypothesis 2**: The salience of renewable energy content in national party manifestos increases the more pro-decentralization the party is.

## Results and discussion

In June 2016, the manifestos of In Common We Can (ECP), Commitment (CMP), and In Tide (EM) featured well-developed, standalone sections on both climate change and energy transition. Citizens (Cs), while having a shorter section, also included a well-articulated part on climate change with a subsection on sustainable energy. Together We Can (UP), Canarian Coalition— Nationalist Canary Party (CC-PNC), the Spanish Socialist Workers’ Party (PSOE), and the People’s Party (PP) also dedicated entire segments to energy issues. While UP included clearer measures on climate change within a broader section devoted to environmentally related topics, the PSOE, Cs, and the PP addressed these issues more sporadically, essentially within their energy sections. The ERC’s manifesto showed a similar trend, approaching both topics in a more general context. The Democratic Convergence of Catalonia (CDC) and the Basque Nationalist Party (EAJ-PNV) addressed these topics within a general environmental section, whereas Unite the Basque Country (EHB) did not include any significant development of them.

In the November 2019 elections, More Country—Equo (MP-E) distinguished itself with a thorough approach to both climate change and the energy transition, addressing each explicitly in the first and second sections of its manifesto, and more generally in the final two sections. Similarly, both CMP and ECP allocated an independent section to each topic. EAJ-PNV, Canarian Coalition—New Canaries (CC-NC) and Together for Catalonia (JxC) also included sections exclusively focused on clean energy. EAJ-PNV and CC-NC used these sections to also cover climate change, while JxC developed this topic more thoroughly in a separate environmental section. In the same vein, UP, ERC, and BNG wrote in length on both issues within broader sections on environmental protection. The PSOE, Cs, and the PP did so to a far lesser degree of refinement. Manifestos from Candidacy of Popular Unity (CUP), EHB, the Regionalist Party of Cantabria (PRC), and Teruel Exists (TE) generally addressed related topics rather than focusing on climate and energy directly, which nonetheless stand out proportionally due to the brevity of these documents. Sum Navarre (NA+) made only scant mentions of these issues throughout their texts, and VOX did not address them at all.

By July 2023, Unite had included two distinct sections on climate change and the energy transition. BNG, the ERC, the PSOE, and the PP each dedicated a section to the second topic and addressed the first one within a broader environmental context. EAJ-PNV, JxC, and EHB also had similar sections on energy, with climate change mentioned transversally across their texts. Without specific sections on any of them, we find mentions of both topics among the measures proposed by the Canarian Coalition (CC), and less often on renewable energy in those of Navarrese People’s Union (UPN) and VOX. Based on the structural evolution of the manifestos studied, there is no discernible pattern in the overall emphasis they place on the analyzed issues. For instance, although political parties are more frequently addressing climate change within broader environmental sections, these areas have gradually become more extensive and comprehensive over time. Therefore, to more accurately assess the significance of climate change in the Spanish elections of June 2016, November 2019, and July 2023, I conducted a quantitative study based on the two variables designed specifically for this purpose.

Table 1 (and Supplementary Figs. 1–3) presents the percentage of content related to each category of the ‘climate code’ variable by political party. The results indicate a significant focus on ‘pro-climate’ content in the manifestos of the political parties studied, with higher figures observed in the 2019 elections. In 2016, CMP, UP, EM, and Cs stood out for assigning greater importance to this category in their manifestos. In 2019, the MP-E coalition dominated, thanks largely to the participation of EQUO, whose environmentalist stance is notable in a country where green policies have traditionally been non-existent at the central level (McFall 2012). Behind this alliance, CMP, EHB, TE, UP, and EAJ-PNV also stand out. Turning to the 2023 elections, parties such as Unite, which filled the ideological space left by UP in previous elections, EAJ-PNV, BNG, and the PSOE were the most vocal on ‘pro-climate’ issues. It is noteworthy that VOX and the PP occupy prominent positions in terms of ‘anti-climate’ content in their manifestos, although they are sometimes surpassed by the PRC and TE in 2019, or UPN in 2023.

**Table 1:** Share of quasi-sentences delivered in party manifestos, by climate code and political party (%)

Climate code	UP	Unite	MP-E	PSOE	Cs	PP	VOX	CUP^a^	ECP^a^	ERC^a^	CDC/JxC^a^	EHB^a^	EAJ-PNV^a^	EM/BNG^a^	CC-PNC/CC-NC/CC^a^	NA+/UPNª	PRC^a^	TE^a^	CMP^a^
*2016*																			
Pro climate policy	12.5	–	–	6.5	8	5.1	–	–	12.6	6.4	7.6	2.6	8	9.5	5.2	–	–	–	21.2
Anti climate policy	0.7	–	–	0.3	1	3.5	–	–	0	0.6	1.2	1	0.8	0	6.3	–	–	–	0.1
*2019*																			
Pro climate policy	17.2	–	78.3	6.3	7.5	7.6	2.8	13.5	7.3	8	9.8	41.5	16.0	8.5	8.9	2.8	13.1	29.4	30.7
Anti climate policy	0	–	0	0.2	2.6	5.6	1.7	0	0.2	0.4	1.2	0	0.9	0	3.2	1	13.9	20.6	0.3
*2023*																			
Pro climate policy	–	21.7	–	13.9	–	6.2	3.2	–	–	12.3	10.8	7.8	18	14.9	12.4	10.5	–	–	–
Anti climate policy	–	0.2	–	1.3	–	2.8	8	–	–	0.1	0.9	0	0.7	1	3.7	6.3	–	–	–

^a^Non-Statewide Parties (NSWPs) Source: scores based on authors’ own measurements (2024).

When it comes to the study of specific ‘pro-climate’ policy subcategories, further variations exist (Table 2 and Supplementary Figs. 4–6). The results of the second, more detailed assessment of ‘pro-climate’ policy importance indicate that the political parties in question generally place greater emphasis on ‘pro-environment’ content in their manifestos, which is logical given that it is the most comprehensive category within the variable it belongs to, followed by ‘pro-lower carbon transport’, ‘pro-renewable energy’, and ‘pro-carbon sinks’. In particular, CMP, UP, and Cs placed notable emphasis on the three aforementioned categories in 2016. Turning to 2019, UP shared this focus with MP-E, which also paid significant attention to these categories, whereas EHB, for instance, almost exclusively focused on ‘waste’ and ‘agriculture and food’. In addition, it is worth noting the figures of other parties, such as CMP, which place special emphasis on ‘pro-energy efficiency’. In 2023, the shift in ideological competition space from UP to Unite is reflected in Unites’ greater emphasis on ‘pro-climate’ categories similar to those previously advocated by UP. It is also worth highlighting the significant emphasis placed on ‘renewable energies’ by EAJ-PNV and BNG.

**Table 2:** Share of quasi-sentences delivered in party manifestos, by pro-climate policy subcategory and political party (%)

Climate code subcategory	UP	Unite	MP-E	PSOE	Cs	PP	VOX	CUP^a^	ECP^a^	ERC^a^	CDC/JxC^a^	EHB^a^	EAJ PNV^a^	EM/BNG^a^	CC-PNC/ CC-NC/CC^a^	NA+/UPN^a^	PRC^a^	TE^a^	CMP^a^
2016																			
Pro-climate categories																			
Pro-Environment	1.7	–	–	2.6	1.4	1.1	–	–	4.4	3.1	1.8	0	3	2.5	0.4	–	–	–	5.1
Pro-renewable energy	2.1	–	–	1	1.8	0.5	–	–	1.2	1	1	3.6	2.6	2	3	–	–	–	3.8
Pro-lower carbon transport	3.2	–	–	0.3	1.2	0.7	–	–	2	1	1.9	0	1.3	1.2	0.2	–	–	–	4
Pro-energy efficiency	0.8	–	–	0.7	0.6	1.3	–	–	0.9	0.1	0.6	0	0.4	0.5	0.4	–	–	–	3.3
Pro-carbon sinks	3.1	–	–	0.6	1.3	0.6	–	–	0.8	0.3	1.3	0	0.4	1	0.2	–	–	–	1.3
Planning	0.6	–	–	0.5	0.5	0.2	–	–	0.3	0	0.2	0	0.2	0.5	0.1	–	–	–	0.4
Agriculture & food	0.5	–	–	0.3	0.2	0.7	–	–	1	0.2	0.3	0	0	1.2	0.4	–	–	–	0.9
Waste	0.5	–	–	0.4	1	0.1	–	–	1.3	0.3	0.6	0	0	0.5	0.2	–	–	–	2.1
Anti-growth	0	–	–	0.1	0	0	–	–	0.3	0.1	0	0	0	0	–	–	–	–	0
Anti-climate categories																			
Pro-roads	0.4	–	–	0	0	0.1	–	–	0	0.4	0.1	0	0	0	0	–	–	–	0
Pro-aviation & shipping	0.1	–	–	0.1	0	0.1	–	–	0	0	0.1	0	0	0	4.4	–	–	–	0
Pro-fossil fuel	0.2	–	–	0	0	0.1	–	–	0	0	0	0	0	0	0	–	–	–	0
Pro-growth	0.1	–	–	0.2	0.6	1.4	–	–	0	0	0.4	0	0.8	0	0.4	–	–	–	0
Anti-taxes	0	–	–	0	0	0.4	–	–	0	0	0.1	0	0	0	0	–	–	–	0
Pro-tourism	0	–	–	0	0.4	0.9	–	–	0	0	0.1	0	0	0	1.2	–	–	–	0.1
Pro-global free trade	0	–	–	0	0	0	–	–	0	0.1	0.4	0	0	0	0	–	–	–	0
Pro-intensive agriculture	0	–	–	0.1	0	0.5	–	–	0	0	0	0	0	0	0.2	–	–	–	0
Anti-regulation	0	–	–	0	0	0	–	–	0	0	0	0	0	0	0	–	–	–	0
Anti-climate (other)	0	–	–	0	0	0	–	–	0	0	0	0	0	0	0	–	–	–	0
2019																			
Pro-climate categories																			
Pro-environment	3.8	–	34.8	3.7	1.8	2.1	0	9.9	5	3	2.8	4.1	3.3	2.1	2.4	1.1	0	1.5	8.7
Pro-renewable energy	1.5	–	7.2	0.4	0	0	0.6	1.8	0.8	1	1.5	0.3	4.4	1.4	3.2	1	0	5.9	5
Pro-lower carbon transport	4.8	–	11.4	0.4	0	0.4	1.1	0	0.6	1.3	2.3	0	2.7	0.9	0.8	0.7	11.5	13.2	3.7
Pro-energy efficiency	1.5	–	2.7	0.4	0	0.4	0	0	0.2	0.3	1.1	0	2.9	0.2	0.3	0.7	0.8	2.9	5.3
Pro-carbon sinks	2	–	0.9	0.5	0	0.4	0.6	0.9	0.1	0.5	0.9	0	0.1	3	0	0	0.8	5.9	1.6
Planning	0	–	4.3	0.2	0	0	0	0	0.1	0.4	0	0	0.9	0	0	0	0	0	0.7
Agriculture & food	3.1	–	7.9	0.5	0	0	0.6	0	0	0.6	0.6	21.6	0.3	0	0.3	0.4	0	0	2.3
Waste	0.3	–	8.7	0.2	0	0	0	0	0.2	0.3	0.6	14.3	1.2	0	2.1	0	0	0	3
Anti-growth	0	–	0.3	0	0	0	0	0.9	0.4	0.7	0	0.7	0	0.9	0	0	0	0	0.1
Anti-climate categories																			
Pro-roads	0	–	0	0	0	0	0	0	0	0	0.2	0	0	0	0	0.4	8.2	20.6	0.1
Pro-aviation & shipping	0	–	0	0	0	0	0	0	0	0	0.3	0	0	0	2.6	0	5.7	0	0.1
Pro-fossil fuel	0	–	0	0.1	0	0	0	0	0	0	0	0	0	0	0	0	0	0	0
Pro-growth	0	–	0	0.1	0	1.1	0.6	0	0.1	0.1	0.7	0	0.6	0	0	0	0	0	0
Anti-taxes	0	–	0	0	0	0	0.6	0	0	0	0.1	0	0	0	0	0.7	0	0	0
Pro-tourism	0	–	0	0	0	0	0	0	0	0.1	0	0	0.1	0	0	0	0	0	0.2
Pro-global free trade	0	–	0	0	0	0.4	0	0	0	0	0	0	0	0	0	0	0	0	0
Pro-intensive Agriculture	0	–	0	0	0	0.4	0	0	0	0.1	0	0	0.1	0	0.5	0	0	0	0
Anti-regulation	0	–	0	0	0	0	0	0	0	0	0	0	0	0	0	0	0	0	0
Anti-climate (other)	0	–	0	0	0	0	0	0	0	0	0	0	0	0	0	0	0	0	0
2023																			
Pro-climate categories																			
Pro-Environment	–	6.3	–	4.9	–	0.8	0.9	–	–	4.1	1.9	2.4	2.9	1.6	3.7	1.1	–	–	–
Pro-Renewable Energy	–	2.1	–	1.1	–	0.8	0.5	–	–	1.7	2.1	1.8	8.6	3.7	1.9	3.2	–	–	–
Pro-lower carbon transport	–	2.5	–	3	–	0.6	0.6	–	–	3.6	4.2	2.4	3.4	3	2.5	3.2	–	–	–
Pro-energy efficiency	–	1.2	–	0.8	–	0.8	0.5	–	–	0.3	0.4	0.6	0.7	0.2	0.6	1.1	–	–	–
Pro-carbon sinks	–	3.2	–	1.1	–	1.2	0.6	–	–	0.6	1.4	0.6	0.5	4.8	3.1	1.1	–	–	–
Planning	–	1.7	–	0.9	–	0.5	0	–	–	0.3	0	0	0.4	0.8	0	0	–	–	–
Agriculture & food	–	3.1	–	1	–	1.4	0.1	–	–	0.5	0.4	0	1.3	0.5	0.6	0	–	–	–
Waste	–	1.4	–	0.7	–	0.2	0.1	–	–	0.7	0.5	0	0.4	0.3	0	0	–	–	–
Anti-growth	–	0.1	–	0	–	0	0	–	–	0.4	0	0	0	0	0	0	–	–	–
Anti-climate categories																			
Pro-roads	–	0	–	0.1	–	0	0.1	–	–	0	0.1	0	0	0.6	0	4.2	–	–	–
Pro-aviation & shipping	–	0	–	0.2	–	0.1	0	–	–	0	0.3	0	0.1	0.2	3.7	0	–	–	–
Pro-fossil fuel	–	0	–	0	–	1.3	1.2	–	–	0	0	0	0	0	0	0	–	–	–
Pro-growth	–	0.1	–	0.8	–	1.3	0.4	–	–	0.1	0	0	0.6	0	0	0	–	–	–
Anti-taxes	–	0	–	0	–	0.5	1.4	–	–	0	0.4	0	0	0	0	1.1	–	–	–
Pro-tourism	–	0	–	0.3	–	0.8	0.3	–	–	0.1	0	0	0	0	0	0	–	–	–
Pro-global free trade	–	0	–	0	–	0	0	–	–	0	0	0	0	0	0	0	–	–	–
Pro-intensive Agriculture	–	0	–	0	–	0.2	1.5	–	–	0.1	0	0	0	0.2	0	1.1	–	–	–
Anti-regulation	–	0	–	0	–	0	0.6	–	–	0	0.1	0	0	0	0	0		–	–
Anti-climate (other)	–	0	–	0	–	0	2.5	–	–	0	0	0	0	0	0	0		–	–

^a^Non-Statewide Parties (NSWPs) Source: scores based on authors’ own measurements (2024).

The most notable ‘anti-climate’ categories in 2019 include ‘pro-growth’, ‘pro-roads’, ‘pro-aviation and maritime transport’, and, to a lesser extent, ‘anti-taxes’, with ‘pro-intensive agriculture’ and ‘pro-fossil fuels’ gaining momentum from 2023 onwards. While in 2016, it was the PP that dominated this aspect due to its focus on ‘pro-growth’ issues, it shared its leadership in this category in 2019 with VOX, JxC, and EAJ-PNV. Interestingly, driven by the specific needs of the regions they represent, CC-NC and TE have begun to excel in their use of ‘pro-aviation and maritime transport’ and ‘pro-roads’, respectively. A similar trend persists in subsequent elections, with the most notable development being the increasing use of ‘pro-fossil fuels’ content by PP and VOX.

**Fig. 1:**
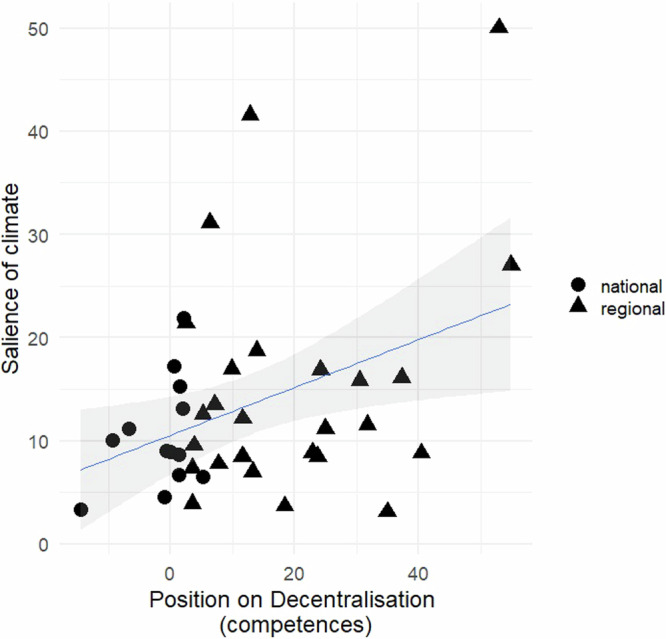
Relationship between a party’s position on decentralization and the salience of climate change in its manifesto. *Source*: The scores on the salience of climate are calculated from the data shown in Table 1. The scores on decentralization are based on Supplementary Table 1. Legend: The variable ‘salience of climate’ accounts for the total percentage of quasi-sentences that each party manifesto dedicates to ‘pro-‘ and ‘anti-‘ climate content. ‘Pro-climate’ quasi-sentences refer to those that promote policies aimed at reducing GHG emissions or increasing GHG sinks, while ‘anti-climate’ quasi-sentences refer to those that advocate for policies leading to increased GHG emissions or reduced GHG sinks. The variable ‘position on decentralization’ reflects the difference between the percentage of quasi-sentences that support decentralization and those that oppose it in each party’s manifesto. Parties represented by triangles belong to the Non-Statewide Party (NSWP) category, while parties represented by circles belong to the Statewide Party (SWP) category.

**Fig. 2:**
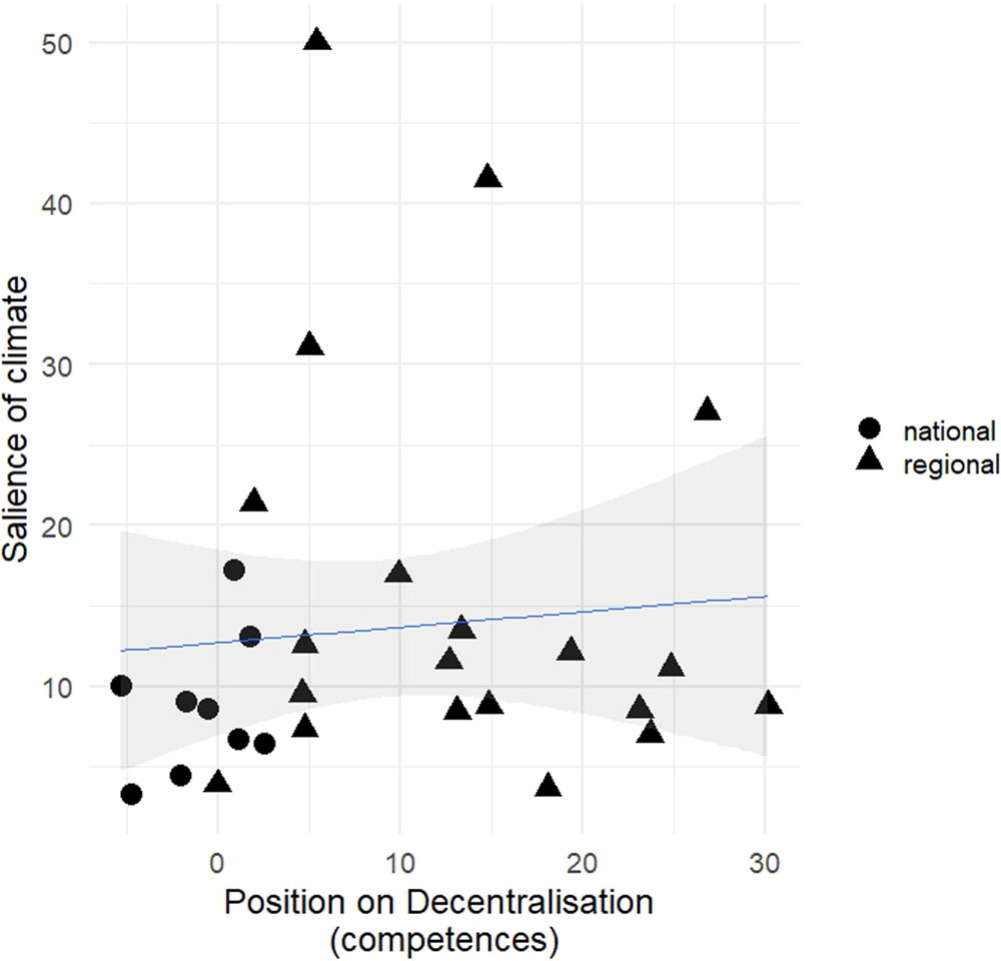
Relationship between a party’s position on decentralization based on MRG/CMP/MARPOR and the salience of climate change in its manifesto. *Source*: The scores on the salience of climate are calculated from the data shown in Table 1. The scores on decentralization are based on the Manifesto Data Collection (MRG/CMP/MARPOR)^60^^.^ Legend: The variable ‘salience of climate’ measures the percentage of quasi-sentences that each party manifesto dedicates to ‘pro-‘ and ‘anti-‘ climate content. ‘Pro-climate’ quasi-sentences refer to those that promote policies aimed at reducing GHG emissions or increasing GHG sinks, while ‘anti-climate’ quasi-sentences refer to those that advocate for policies leading to increased GHG emissions or reduced GHG sinks. The variable ‘position on decentralization’ reflects the difference between the ‘Decentralization’ and ‘Centralization’ variables from the MRG/CMP/MARPOR^60^. Parties represented by triangles belong to the Non-Statewide party (NSWP) category, while parties represented by circles belong to the Statewide party (SWP) category.

The percentages shown in Tables 1 and 2 provide initial support for hypotheses 1 and 2. While these figures highlight the increased emphasis on climate change among left-wing parties, we find notable exceptions, such as the center-right parties CDC/JxC in 2016 and 2019, as well as EAJ-PNV in 2019 and 2023. This indicates that although political ideology likely influences climate policy preferences, it is true that NSWPs—compared to most SWPs, including some left-leaning ones like the PSOE in 2016 and 2019—pay greater attention to climate issues. To confirm or reject the first hypothesis it is necessary to examine the link between decentralization and climate policy preferences (Figs. 1 and 2). The data indicate that parties exhibiting a stronger pro-decentralization stance along the center-periphery axis tend to place greater importance on climate content in their national election manifestos. Similarly, the results in both figures reveal that the NSWPs, which are the actors most in favor of decentralization, consistently exhibit the highest values regarding the importance of climate issues.

This finding is reflected in the manifestos of notable pro-decentralization parties, which express the regions’ desire to manage the resources and competences related to climate change:

*‘Transfer the proceeds from the auction of emission rights to the Government of Catalonia for them to be allocated to the fight against climate change’* ^46^

‘*Demand for the resources allocated to research and the implementation of policies for mitigating and adapting to climate change to be distributed among the territories of the ACs’*^47^

*‘In terms of jurisdiction, we advocate for the transfer of all environmental competencies to Galicia’* ^48^

*‘We will provide municipalities with the necessary resources for reducing greenhouse gas (GHG) emissions’* ^49^

**Fig. 3:**
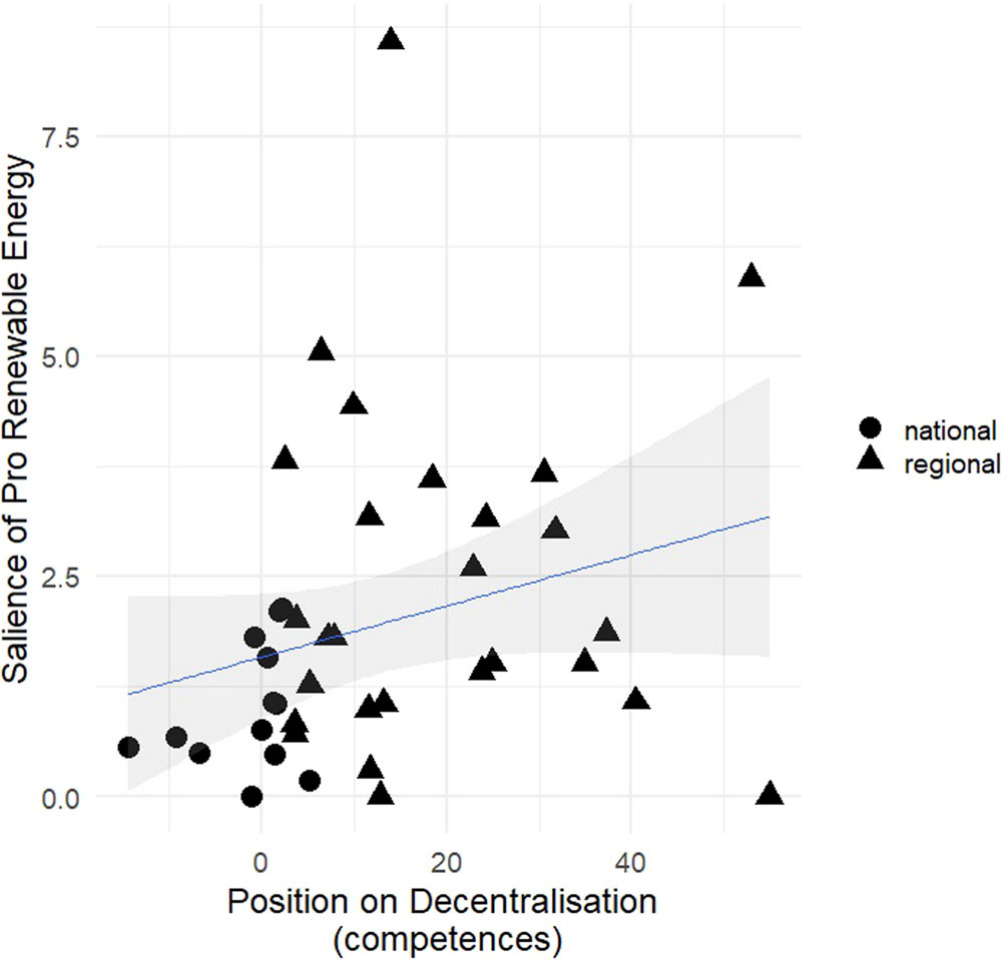
Relationship between a party’s position on decentralization and the salience of pro-renewable energy in its manifesto. *Source*: The scores on the salience of renewable energy are calculated from data shown in Table 2. The scores on decentralization are based on Supplementary Table 1. Legend: The variable ‘salience of pro-renewable energy’ measures the percentage of quasi-sentences that each party manifesto dedicates to advocating for the shift in the production, distribution, and consumption of electricity from conventional, fossil fuel-based sources to sustainable and renewable energy sources. The variable ‘position on decentralization’ reflects the difference between the percentage of quasi-sentences that support decentralization and those that oppose it in each party’s manifesto. Parties represented by triangles belong to the Non-Statewide party (NSWP) category, while parties represented by circles belong to the Statewide party (SWP) category.

**Fig. 4:**
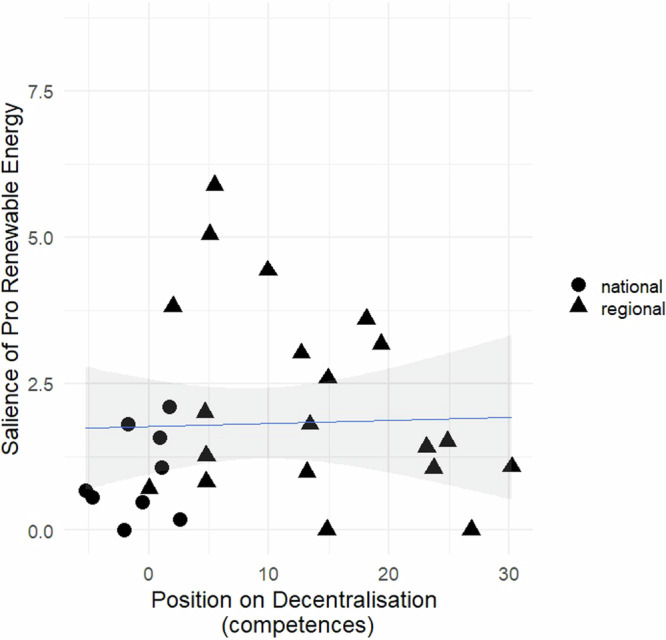
Relationship between a party’s position on decentralization based on MRG/CMP/MARPOR and the salience of pro-renewable energy in its manifesto. *Source*: The scores on the salience of renewable energy are calculated from data shown in Table 2. The scores on decentralization are based on MRG/CMP/MARPOR^60^. Legend: The variable ‘salience of pro-renewable energy’ measures the percentage of quasi-sentences that each party manifesto dedicates to advocating for the shift in the production, distribution, and consumption of electricity from conventional, fossil fuel-based sources to sustainable and renewable energy sources. The variable ‘position on decentralization’ reflects the difference between the ‘Decentralization’ and ‘Centralization’ variables from the MRG/CMP/MARPOR^60^. Parties represented by triangles belong to the Non-Statewide party (NSWP) category, while parties represented by circles belong to the Statewide party (SWP) category.

To test the second hypothesis, I analyze the association between the position on decentralization and the salience of ‘pro-renewable energy’ (Figs. 3 and 4). Especially noteworthy is the first figure, which is more comprehensive as it includes data from the 2023 elections; in particular, NSWPs like EAJ-PNV, BNG, and UPN dominated the last-mentioned category. This (more clearly) evinces the existence of a positive relationship between the two variables: Political parties that adopt a more pro-decentralization stance tend to place greater emphasis on pro-renewable energy in their national election manifestos. Again, the results in both figures reveal that the NSWPs generally exhibit the highest values concerning the importance of the RET.

This aligns with the demands expressed through various quasi-sentences identified in the manifestos. In these, the NSWPs demonstrate awareness of their regions’ potential for renewable energy generation and the opportunities it provides them:

*‘The potential for renewable energy generation, thanks to the abundant resources (wind and sun), along with the high investor interest, should be leveraged to develop renewable installations that meet current electrical needs, future needs driven by the electrification of other types of consumption, and for the production of green hydrogen’* ^50^

*‘The set of energy policies and climate change adaptation measures harbor significant employment opportunities and the capacity to stimulate the economy’* ^51^

Similarly, they often emphasize the detrimental impact of the state’s recentralizing and extractive role regarding this issue:

*‘The actions of the central government regarding the energy sector in recent years have been characterized by a clear process of recentralizing some of the competencies granted to the ACs’* ^52^

*‘The BNG will advocate in all areas, and particularly in the Cortes, for overcoming a dependent and extractive model that has turned Galicia into a provider of raw materials and energy, with extremely high social and environmental costs’* ^48^

*‘The reform of the electricity sector driven by the State has negatively impacted electricity generation installations based on cogeneration, renewable energies, and waste. In Catalonia, 3866 installations have seen their premiums reduced from 732 million euros to 543 million euros, resulting in a loss of incentives amounting to 189 million euros and a reduction in remuneration of more than 25%’* ^47^

Accordingly, the NSWPs consistently advocate for decentralized renewable generation:

*‘It is necessary to defend our right to choose our own energy model, to contribute to the fight against the climate crisis, and to do so in a way whereby the productive role benefits the social and economic interests of Galicia while safeguarding the preservation of biodiversity’* ^48^

*‘In particular, the State will be urged to implement specific measures to accelerate the energy transition in the Canary Islands (considering that in isolated and insular territories such as the Canary Islands, it is more challenging to undertake the necessary changes for the new energy paradigm)’* ^53^

*‘The energy transition towards a system where demand is predominantly met by renewable energies implies a progressive electrification of consumption and increasingly decentralized generation—one of smaller size and that is closer to consumption points’* ^50^

‘*The energy transition towards a 100% renewable model must be based on the decentralized production and management of energy, which means that the role of local administrations—alongside citizens and the cooperative movement—is crucial in a self-production-based model.’*^49^

‘*We must shift towards a decarbonized model without nuclear power plants – a decentralized one in which renewable energy is self-produced locally—to achieve the goal of zero GHG emissions by 2050’*^54^

The evidence gathered from the data confirms the two hypotheses of this study. Given the consistent positive correlations observed, it is reasonable to suggest that political parties with stronger pro-decentralization inclinations tend to prioritize climate change and the RET in Spanish national elections. The positive relationship between devolution and the advancement of climate policy and the RET supports a decentralized approach to both issues as decentralization raises the efficiency and legitimacy of climate management.

To this end, lower levels of government empowered within decentralized systems are best equipped to tailor their actions to local conditions, thereby leveraging local knowledge, skills, and resources to promote effective climate policies^5^. Such an approach also enhances responsiveness and efficiency in addressing region-specific needs while balancing land use and environmental considerations of the RET^14,18^. Moreover, decentralization plays a crucial legitimizing role by granting autonomy to regional entities in developing and implementing climate policies^5,9^. It also promotes models of ‘energy democracy’, where active citizens engage in renewable energy production and consumption^21,22^. Lastly, subnational governments can play a complementary or compensatory role in national climate action^3,12,13^. Specifically, they can contribute to realizing the RET by decreasing their energy dependence on the central government and developing their own renewable energy strategies and policies when they possess the necessary jurisdictional capacity^15^.

On a different note, NSWPs may have additional, territorial motivations that partly explain the presented findings. While it would exceed this study’s scope to compare the specific roles of these parties versus Statewide parties (SWP)—some of which, in the Spanish context, sometimes adopt pronounced pro-decentralization positions due to the significance of this divide—NSWPs assert leadership in both areas. As highlighted in the existing literature on Green Nationalism in regional elections^45^, their leadership on climate and renewable energy issues at the central level would be motivated by a desire to challenge the institutional *status quo* of the State and enhance the autonomy of the regions they represent^37–39^. Indeed, as shown by the quasi-sentences quoted above, NSWPs often invoke climate change to pursue greater economic and/or political autonomy at the substate level^3^.

In accordance with the above, the evidence in Figs. 3 and 4 shows that pro-decentralization parties are interested in acquiring higher autonomy with respect to energy-related issues. This interest would stem from the potential control that regions could wield over the growing network of renewable energy installations during the RET. In turn, the NSWPs could increase their political clout with regard to energy policy and, at the same time, decrease the dominant position of the state as the leading energy supplier.

This paper contributes to the theoretical development of Climate Federalism, as empirical evidence from the analysis illustrates that the underlying pro-decentralization orientations of political parties are likely to put greater emphasis on climate change and the RET, for which reason they often assume climate leadership in multilevel democracies. The findings also highlight the strategic role of subnational politics in shaping national climate agendas, reinforcing the notion of Green Nationalism as a critical framework for understanding climate leadership not only at the regional level but also at the national one. This paper thus fills the gap between decentralization and climate policy preferences by informing academic debates on how the support of climate policy can be enhanced under multilevel governance systems through interparty competition by subnational actors seeking regional self-government.

## Methods

The methodology employed in this article began with an initial qualitative analysis to assess the extent to which the issue of climate change is integrated into the structure of the electoral manifestos from the main political parties that competed in three significant Spanish general elections: June 2016, November 2019, and July 2023. For the June 2016 elections, the analysis covered the manifestos of the PP, PSOE, Cs, UP, EAJ-PNV, EHB, EM, ERC, CDC, ECP, CC-PNC, and CMP. For the November 2019 elections, the parties included were the PP, PSOE, Cs, UP, MP-E, VOX, CUP, JxC, ECP, ERC, EAJ-PNV, EHB, BNG, CC-NC, TE, PRC, NA+, CMP. For the July 2023 elections, the parties analyzed were the PP, PSOE, Cs, Unite, VOX, JxC, ECP, ERC, EAJ-PNV, EHB, BNG, CC, and UPN.

This selection of parties highlights Spain’s two-dimensional national party system, characterized by prominent left-right and center-periphery cleavages. For instance, there are both SWPs and NSWPs, on the left and the right, which advocate for various degrees of regional autonomy from the Spanish State^55^ (Table 3). The SWPs, such as the PP, PSOE, Unite, MP-E, Cs, and VOX, operate across the national territory and focus on policies for Spain as a whole. In contrast, the NSWPs, including EAJ-PNV, EHB, ERC, CDC, JxC, CUP, ECP, EM, BNG, CC-PNC, CC-NC, CC, NA+, UPN, TE, and CMP, represent specific regions and advocate for varying degrees of regional autonomy. While NSWPs often dominate the regional political landscape, either governing (e.g., EAJ-PNV) or leading the opposition (e.g., EHB), they typically play supportive roles at the national level, becoming key allies for minority governments^56,57^.

**Table 3:** Classification of NSWPs and SWPs in Spain by election (2016–2023)

	Statewide parties	Non-statewide parties
June 2016	PP, PSOE, Cs, UP	EAJ-PNV, EHB, CDC, ERC, EM, ECP, CC-PNC, CMP
November 2019	PP, PSOE, Cs, UP, MP-E, VOX	EAJ-PNV, EHB, JxC, CUP, ERC, BNG, ECP, CC-NC, CMP, TE, PRC, NA+
July 2023	PP, PSOE, Cs, Unite, VOX	EAJ-PNV, EHB, JxC, ERC, BNG, ECP, CC, UPN.

*Source*: Own elaboration (2024).

Following the qualitative stage, I conducted a quantitative analysis using quasi-sentences as the unit of observation, in line with the method described by Schmitt and Wüst^58^. This approach resulted in a hand-coded database containing a total of 49,816 quasi-sentences. This second phase of the study adopted a climate policy focus, in line with Carter et al.‘s^59^ meticulous conceptualization and operationalization of this concept. This approach addressed shortcomings in previous measurements, which predominantly concentrated on environmental protection (e.g., the Comparative Agendas Project or the Comparative Manifestos Project), by employing four categorical variables. One variable quantified the percentage of quasi-sentences in each political party’s manifesto, categorizing them as ‘pro-climate’, ‘anti-climate’, ‘neutral’, or ‘not sufficiently relevant’ concerning net GHG emissions. ‘Pro-climate’ content advocated policies aimed at reducing GHG emissions or increasing GHG sinks, while ‘anti-climate’ content supported policies leading to increased GHG emissions or reduced GHG sinks. ‘Neutral’ content pertained to net GHG emissions and implied that emissions would remain unchanged. Content classed as ‘not sufficiently relevant’ lacked substantial relevance to net GHG emissions and thus did not fall into any of the other categories.

Subsequently, I devised another variable to offer additional insights into the ‘pro’ and ‘anti’ climate categories and to systematically diversify the content of the measures. This variable divided the ‘pro-climate’ category from the initial variable into more detailed subcategories: ‘Pro-environment’, ‘Pro-climate policy’, ‘Pro-renewable energy’, ‘Pro-lower carbon transport’, ‘Pro-energy efficiency’, ‘Pro-carbon sinks’, ‘Planning’, ‘Agriculture and food’, ‘Waste’, and ‘Anti-growth’. Similarly, the ‘anti-climate’ quasi-sentences were encoded into additional fine-grained subcategories: ‘Pro-roads’, ‘Pro-aviation and shipping’, ‘Pro-fossil fuel’, ‘Anti-environment’, ‘Anti-climate (other)’, ‘Pro-growth’, ‘Anti-environmental taxes’, ‘Pro-tourism’, ‘Pro-global free trade’, ‘Pro-intensive agriculture’, and ‘Anti-regulation’. Later, the proportions explained by the first variable were used as the dependent variable in a salience estimation that factored in the sum of ‘pro-climate’ and ‘anti-climate’ content per political party’s manifesto. To gauge the content dedicated to the RET, this research focused on the ‘pro-climate’ subcategory ‘pro-renewable energy’. It includes the percentage of quasi-sentences over the total of each party manifesto that speaks in favor of shifting the production, distribution, and consumption of electricity from conventional, fossil-fuel-based sources to sustainable and renewable energy sources. This category hence encompasses the content related to the electricity subsector within the energy transition.

When examining the independent variable, I analyzed center-periphery preferences using two distinct positional measures. Given that the main databases lacked approaches addressing this cleavage for the parties that competed in the 2023 Spanish general elections, I constructed the first measure by calculating the difference between the hand-coded quasi-sentences for and against decentralization in each party’s manifesto. These measures were respectively inspired by the content captured by the ‘Decentralization’ and ‘Centralization’ variables from the MRG/CMP/MARPOR^60^. The first of these variables captures the proportion of quasi-sentences advocating for federalism or the decentralization of political and/or economic power, while the second focuses on the proportion of quasi-sentences that oppose political decision-making at lower political levels, support a unitary government, or advocate for greater centralization in political and administrative processes. Additionally, despite only having data for the parties that competed in the June 2016 and November 2019 elections, I introduced a supplementary positional measure. This was based on the difference between the ‘Decentralization’ and ‘Centralization’ variables from the MRG/CMP/MARPOR^60^, providing a contrasting perspective. Finally, I included a variable to differentiate between SWPs and NSWPs, a distinction consistently represented in all graphs throughout the results section.

After obtaining all the measurements, the scores for each party were plotted, and the correlation between the variables of interest was analyzed descriptively. In the final phase of the analysis, I excluded the political party MP-E due to its unusually high values in climate salience, which deviate significantly from the overall data pattern and complicate the interpretation of the descriptive results and any underlying patterns. I also incorporated key quasi-sentences— identified through a qualitative analysis of the manifestos—to uncover deeper insights.

## Supplementary information

Supplemental Material

## Data Availability

The data that support the findings of this study are available on request from the corresponding author (J.E.).
