# Supplementary material for: Party preferences for climate policy and the renewable energy transition in Spain’s multilevel democracy
Source: NPJ Clim Action. 2024 Oct 31;3(1):93. doi: 10.1038/s44168-024-00183-5 (PMC11525191; doi:10.1038/s44168-024-00183-5)
Supplement: Supplementary file 1 — Supplemental Material [file 44168_2024_183_MOESM1_ESM.pdf]

<u>Supplementary Information</u>

Supplementary Figure 1. Share of quasi-sentences delivered in party manifestos in the Spanish national elections of 2016, by climate code and political party (percent).

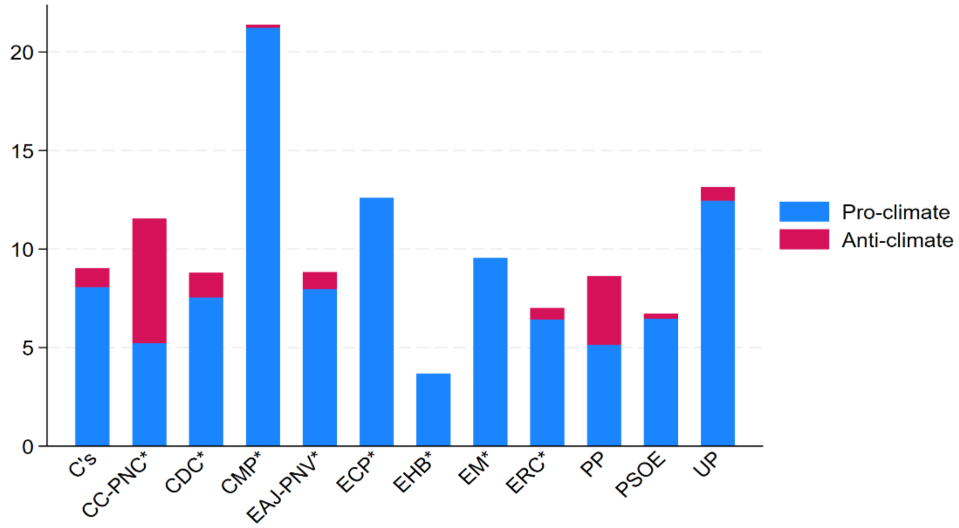

*Non-Statewide Parties (NSWPs)

Scores based on authors' own measurements

Legend: Blue bars show the percentage of quasi-sentences in each political party's manifesto that promote policies aimed at reducing GHG emissions or increasing GHG sinks, while red bars show the percentage of quasi-sentences in each manifesto dedicated to promoting policies that lead to increased GHG emissions or reduced GHG sinks.

Supplementary Figure 2. Share of quasi-sentences delivered in party manifestos in the Spanish national elections of 2019, by climate code and political party (percent).

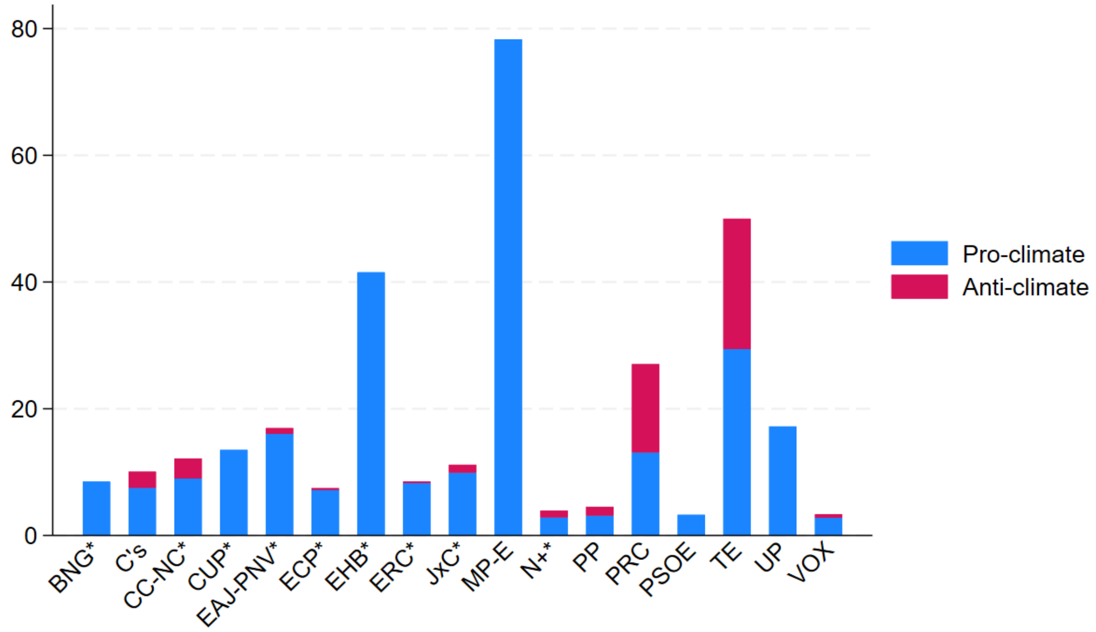

*Non-Statewide Parties (NSWPs)

Scores based on authors' own measurements

Legend: Blue bars show the percentage of quasi-sentences in each political party's manifesto that promote policies aimed at reducing GHG emissions or increasing GHG sinks, while red bars show the percentage of quasi-sentences in each manifesto dedicated to promoting policies that lead to increased GHG emissions or reduced GHG sinks.

Supplementary Figure 3. Share of quasi-sentences delivered in party manifestos in the Spanish national elections of 2023, by climate code and political party (percent).

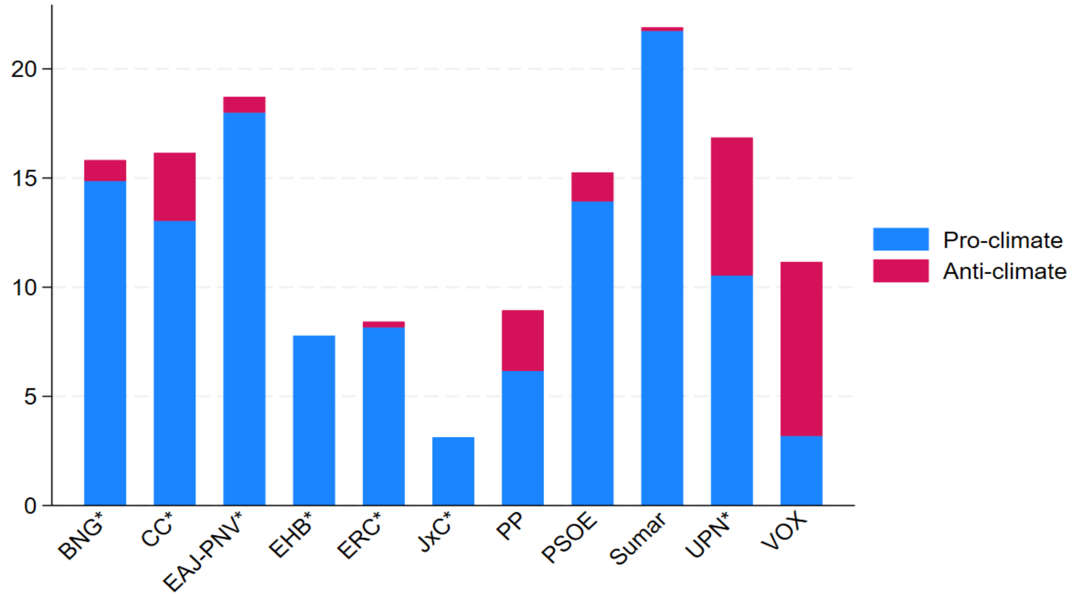

*Non-Statewide Parties (NSWPs)

Scores based on authors' own measurements

Legend: Blue bars show the percentage of quasi-sentences in each political party's manifesto that promote policies aimed at reducing GHG emissions or increasing GHG sinks, while red bars show the percentage of quasi-sentences in each manifesto dedicated to promoting policies that lead to increased GHG emissions or reduced GHG sinks.

Supplementary Figure 4. Share of quasi-sentences delivered in party manifestos in the Spanish national elections of 2016, by climate code and political party (percent).

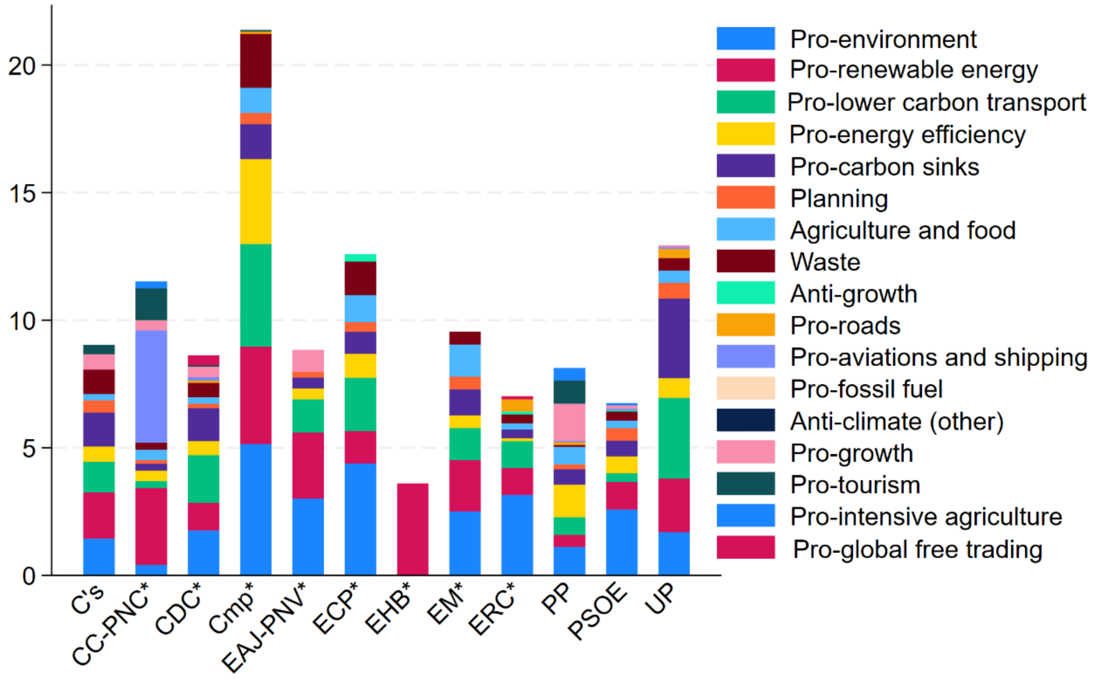

*Non-Statewide Parties (NSWPs)

Scores based on authors' own measurements

Legend: Bars of different colors show the percentage of quasi-sentences in each political party's manifesto dedicated to promoting policies within different 'pro' and 'anti' climate categories. Among the 'pro-climate' categories, we find: 'pro-environment', 'pro-renewable energy', 'pro-lower carbon transport', 'pro-energy efficiency', 'pro-carbon sinks', 'planning', 'agriculture and food', and 'waste'. Among the 'anti-climate' categories, we find: 'pro-roads', 'pro-aviation and shipping', 'pro-fossil fuel', 'anti-climate (other)', 'pro-growth', 'pro-tourism', 'pro-intensive agriculture', and 'pro-global free trade'.

Supplementary Figure 5. Share of quasi-sentences delivered in party manifestos in the Spanish national elections of 2019, by climate code and political party (percent).

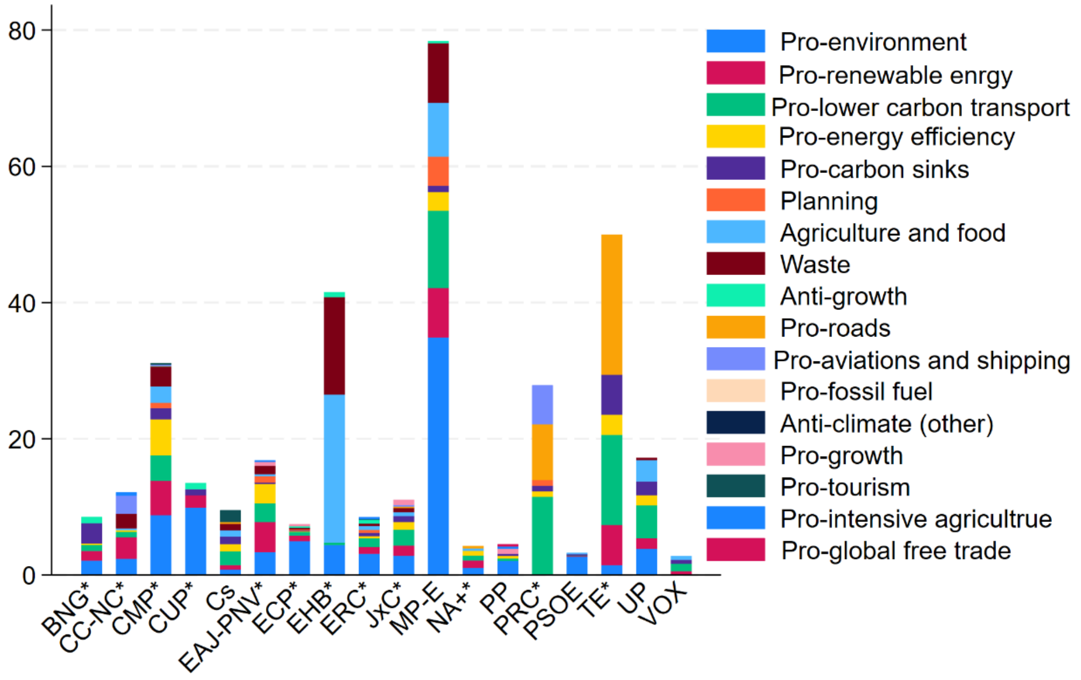

*Non-Statewide Parties (NSWPs)

Scores based on authors' own measurements

Legend: Bars of different colors show the percentage of quasi-sentences in each political party's manifesto dedicated to promoting policies within different 'pro' and 'anti' climate categories. Among the 'pro-climate' categories, we find: 'pro-environment', 'pro-renewable energy', 'pro-lower carbon transport', 'pro-energy efficiency', 'pro-carbon sinks', 'planning', 'agriculture and food', and 'waste'. Among the 'anti-climate' categories, we find: 'pro-roads', 'pro-aviation and shipping', 'pro-fossil fuel', 'anti-climate (other)', 'pro-growth', 'pro-tourism', 'pro-intensive agriculture', and 'pro-global free trade'.

Supplementary Figure 6. Share of quasi-sentences delivered in party manifestos in the Spanish national elections of 2023, by climate code and political party (percent).

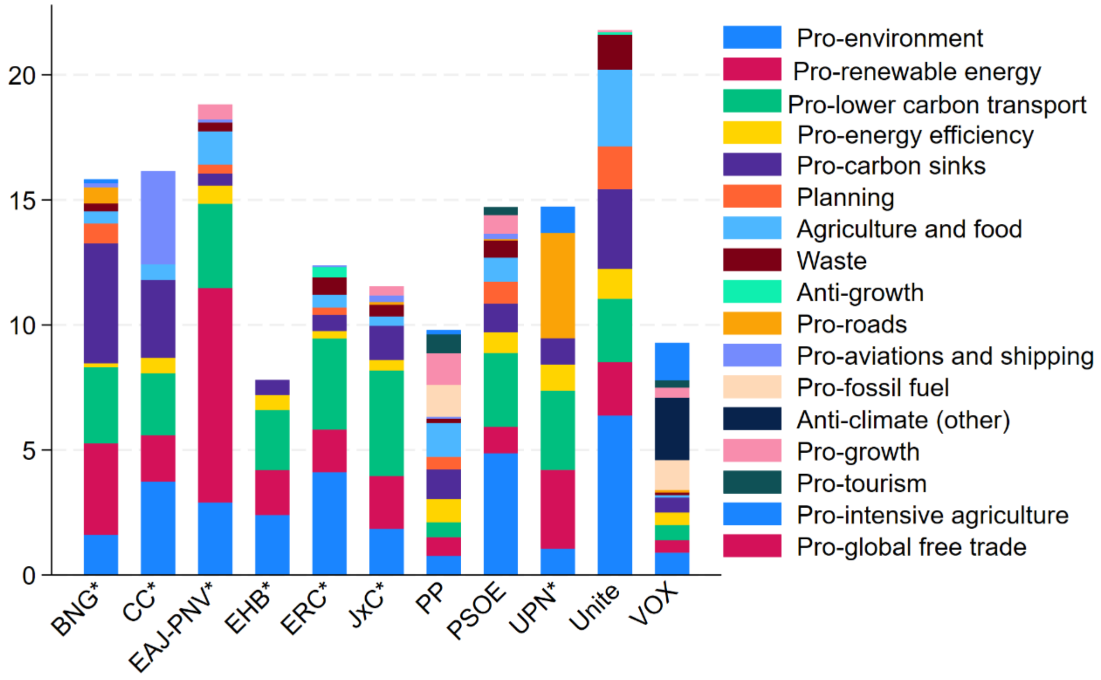

*Non-Statewide Parties (NSWPs)

Scores based on authors' own measurements

Legend: Bars of different colors show the percentage of quasi-sentences in each political party's manifesto dedicated to promoting policies within different 'pro' and 'anti' climate categories. Among the 'pro-climate' categories, we find: 'pro-environment', 'pro-renewable energy', 'pro-lower carbon transport', 'pro-energy efficiency', 'pro-carbon sinks', 'planning', 'agriculture and food', and 'waste'. Among the 'anti-climate' categories, we find: 'pro-roads', 'pro-aviation and shipping', 'pro-fossil fuel', 'anti-climate (other)', 'pro-growth', 'pro-tourism', 'pro-intensive agriculture', and 'pro-global free trade'.

Supplementary Table 1. Position on Decentralization

Election	Political Party	Position on Decentralization
2016	C's	-0.7
2016	CDC	40.4
2016	UP	1.9
2016	PP	1.41
2016	PSOE	1.3
2016	ECP	5.2
2016	ERC	13.2
2016	EHB	18.4
2016	EAJ-PNV	22.8
2016	EM	3.7
2016	CMP	2.5
2016	CC-PNC	31.7
2019	UP	0.6
2019	MP-E	1.8
2019	PP	-1.1
2019	PSOE	5.2
2019	VOX	-14.4
2019	C's	-9.3
2019	BNG	23.7
2019	CC-NC	11.6
2019	EHB	12.8
2019	EAJ-PNV	9.9
2019	ERC	11.5
2019	JxC	25
2019	CUP	7.1
2019	ECP	3.5
2019	NA+	3.5
2019	TE	52.9
2019	PRC	54.9

2019	CMP	6.4
2023	UNITE	2.1
2023	PP	-0.1
2023	PSOE	1.5
2023	VOX	-6.8
2023	BNG	30.5
2023	CC	37.3
2023	EHB	7.8
2023	EAJ-PNV	13.9
2023	ERC	11.4
2023	JxC	34.9
2023	UPN	24.2

* Scores based on authors' own measurements
